# IgG glycan hydrolysis by EndoS inhibits experimental autoimmune encephalomyelitis

**DOI:** 10.1186/1742-2094-9-209

**Published:** 2012-09-03

**Authors:** Mahdia Benkhoucha, Nicolas Molnarfi, Marie-Laure Santiago-Raber, Martin S Weber, Doron Merkler, Mattias Collin, Patrice H Lalive

**Affiliations:** 1 Department of Pathology and Immunology, Faculty of Medicine, University of Geneva, 1211, Geneva, Switzerland; 2 Department of Clinical Neurosciences, Division of Neurology, Unit of Neuroimmunology and Multiple Sclerosis, Geneva University Hospital, 1211, Geneva, Switzerland; 3 Department of Neurology, Technische Universität München, D-08538, Munich, Germany; 4 Division of Clinical Pathology, Geneva University Hospital, 1211, Geneva, Switzerland; 5 Department of Neuropathology, University Medical Center, Georg August University, Göttingen, Germany; 6 Department of Clinical Sciences, Division of infection Medicine, Lund University, SE-221 84, Lund, Sweden; 7 Department of Genetic and Laboratory Medicine, Division of Laboratory Medicine, Geneva University Hospital, 1211, Geneva, Switzerland

## Abstract

Studies in experimental autoimmune encephalomyelitis (EAE), a mouse model of multiple sclerosis, have shown that B cells markedly influence the course of the disease, although whether their effects are protective or pathological is a matter of debate. EndoS hydrolysis of the IgG glycan has profound effects on IgG effector functions, such as complement activation and Fc receptor binding, suggesting that the enzyme could be used as an immunomodulatory therapeutic agent against IgG-mediated diseases. We demonstrate here that EndoS has a protective effect in myelin oligodendrocyte glycoprotein peptide amino acid 35–55 (MOG_35-55_)-induced EAE, a chronic neuroinflammatory demyelinating disorder of the central nervous system (CNS) in which humoral immune responses are thought to play only a minor role. EndoS treatment in chronic MOG_35-55_-EAE did not impair encephalitogenic T cell priming and recruitment into the CNS of mice, consistent with a primary role of EndoS in controlling IgG effector functions. In contrast, reduced EAE severity coincided with poor serum complement activation and deposition within the spinal cord, suggesting that EndoS treatment impairs B cell effector function. These results identify EndoS as a potential therapeutic agent against antibody-mediated CNS autoimmune disorders.

## Introduction

Multiple sclerosis (MS) is a chronic inflammatory disease of the central nervous system (CNS) characterized by immune cell infiltration into the white matter, which in turn produces demyelination, axonal damage, and the neuronal loss that is considered the main cause of permanent neurological deficits. The concept of MS as a purely T-cell-driven autoimmune disease has been challenged by recent studies indicating an important role for B cells in the pathogenesis of the disease [1]. Several key findings suggest a pathogenic role for B cells and antibodies in MS, mainly in association with antibody deposition and complement activation. Intrathecal immunoglobulin G (IgG) synthesis and oligoclonal bands are found in more than 90% of MS patients [2], clonally expanded B cells accumulate in chronic MS lesions and in the CSF of MS patients [3], and B-cell follicle-like structures in the meninges of patients have been identified by histopathology [4]. The results of histopathological analyses indicate that antibodies might have an important role in plaque initiation and demyelination in patients with established MS [5,6]. While the antigen target of these antibodies has yet to be identified [7-11], the presence of intrathecal anti-myelin Ig has been associated with rapid disease progression [1,12].

Experimental autoimmune encephalomyelitis (EAE) in mice closely mimics the inflammatory infiltration, neurological paralytic symptoms, and demyelination observed in MS. In recent studies, the EAE model has been critical in dissecting the different roles that B cells play in the regulation of MS, and has led to new insights into B cell functions in human pathogenesis and a focus on the development of B cell therapeutics. B cell depletion therapy has recently been examined in EAE induced by myelin oligodendrocyte glycoprotein amino acid 35–55 (MOG_35-55_), and confirmed that both protective and pathological B cell functions markedly influence the course of disease [13-16]. Previous studies indicated that congenitally B cell-deficient mice and CD19-deficient mice with reduced B cell function develop a severe non-remitting form of MOG_35-55_-induced EAE [17], suggesting a regulatory role for B cells, most likely in association with the production of IL-10 [14,17]. However, in the MOG_35-55_-induced EAE mouse model, an accumulating body of evidence also indicates a possible contribution of myelin-reactive antibodies to EAE pathogenesis [18]. First, the transfer of anti-MOG serum from MOG_35-55_-immunized mice has been demonstrated to increase the severity of EAE in wild-type (WT) mice [19]; second, significant positive correlations were established between anti-MOG_35-55_ antibody levels and clinical scores, leukocyte infiltration and reactive astrocytosis in the spinal cord of an animal model of EAE achieved with a multiple MOG_35-55_ peptide [20]; and third, mice immunized with MOG_35-55_ peptides developed significant serum levels of anti-MOG antibodies coinciding with clinical EAE severity [21]. These studies imply that MOG peptide-specific antibodies may be pathogenic in this strain, as appears to be the case in several mouse and rat strains [22].

The capacity of anti-MOG antibodies to contribute to an ongoing CNS inflammatory and demyelinating response has been previously demonstrated in models of antibody-augmented EAE [23-26]. However, how such autoantibodies might mediate tissue destruction and demyelination in EAE remains a subject of intense debate [27]. Traditionally, pathogenic antibodies or autoantibodies have been implicated in MS through their ability to mediate their effector function, either via recruitment of the classical complement cascade or through activatory Fc receptor (FcγR)-mediated antibody-directed cell-mediated cytotoxicity (ADCC) [27]. The presence of deposited IgG around the borders of actively demyelinating MS plaques correlates with the presence of activated complement fragments and complexes [5,28,29] and antibodies bound to fragment of myelin within phagocytic cells are found at the sites of demyelination [30,31]. In EAE, the role of complement and Fc receptors in disease pathogenesis remains unclear. Although demyelination was found to be augmented in brains and spinal cords of transgenic mice engineered to produce increased levels of anti-MOG antibodies [26], experiments demonstrated that complement has little or no involvement in the autoantibody-mediated pathology, but that the mechanism for exacerbation involved Fc-mediated effects [32]. However, other data indicate that inoculation of anti-MOG antibodies drastically increases clinical MOG_35-55_-induced EAE in an activatory FcγR-independent yet complement-dependent fashion [33], and that in the Lewis rats, the demyelination potential of MOG-specific antibodies has been shown to correlate with complement-binding ability [34]. The role of complement activation in MOG_35-55_-induced EAE has been debated. While complement-deficient mice [35,36] or expression of the complement inhibitor sCrry attenuates EAE [37], other data indicate that key components of the complement system, such as C1q, C3, C4, and C5a, may not be involved in MOG_35-55_-induced EAE pathogenesis [33,36,38,39].

Regarding the role of autoantibodies in various autoimmune conditions, novel therapeutic agents that directly target pathogenic antibodies have recently been successfully developed. The IgG-degrading enzyme of *Streptococcus pyogenes* (IdeS) prevented an antibody-induced relapse in mice that had chronic arthritis, and delayed the onset and reduced the severity of experimental collagen-induced arthritis (CIA) [40]. Like IdeS, EndoS from *Streptococcus pyogenes* is an immunomodulating enzyme that specifically hydrolyzes glycans from human IgG, and thereby affects antibody effector functions. EndoS has recently been demonstrated as an attractive treatment in autoantibody-mediated autoimmune disease mouse models of CIA [41], lethal IgG-driven immune thrombocytopenic purpura (ITP), autoimmune hemolysis [42,43], and glomerulonephritis [44]. Thus, modulation of IgG glycosylation is a promising strategy to interfere with autoantibody-mediated inflammatory processes, such as those that may take place during the pathogenesis of MS or its animal model, EAE.

In the present study, we evaluated the importance of glycosylation of the IgG molecule by EndoS treatment in MOG_35-55_-induced EAE, the most commonly used model to study the efficacy of potential drugs for the treatment of MS. In this chronic demyelinating disease of the CNS we previously showed that specific antibodies against MOG and nucleic acids (RNA and ssDNA) paralleled disease progression. Here, we observed that IgG deglycosylation by EndoS treatment attenuates MOG_35-55_-induced EAE development in WT mice but not in B-cell-deficient mice, suggesting that antibodies aggravate the clinical course in this EAE model. Histological analysis showed reduced CNS inflammatory lesions and demyelination. Data also revealed significantly reduced serum complement consumption and CNS deposition in EndoS-treated mice versus untreated mice, suggesting a key role for complement in EAE progression. Collectively, this study establishes a role for antibodies in MOG peptide-induced immune-mediated demyelination, a model largely considered to be purely T cell-dependent, and suggests that complement activation may be relevant in the context of antibody-exacerbated EAE.

## Materials and methods

### Ethics statement

Animal experiments described in the present study were approved by the Ethics Committee for Animal Experimentation of the Faculty of Medicine, University of Geneva, Switzerland (protocol ID number: 1005/3537/3).

### Induction of EAE

Acute chronic EAE was induced in 6- to 8-week-old WT or B cell-deficient (μMT KO) C57BL6J female mice, as previously reported [45,46]. Briefly, mice were immunized subcutaneously with 100 μg of MOG_35-55_ peptide (MEVGWYRSPFSRVVHLYRNGK; AnaSpec, USA) emulsified in CFA and supplemented with 200 μg of *Mycobacterium tuberculosis* strain H37RA (Difco, Detroit, MI, USA). The mice received intravenous injections with 300 ng pertussis toxin (Sigma-Aldrich, St Louis, MO, USA) at the time of immunization and 48 h later. Clinical disease was scored daily as follows: 0, no clinical disease; 1, limp tail; 2, impaired righting reflex; 3, hind limb paralysis; 4, moribund; 5, dead. Severely paralyzed mice were afforded easier access to food and water. Each time point presents the average disease score of each group. Statistical significance was assessed using an unpaired Student’s *t*-test.

### EndoS enzyme treatment

EndoS was recombinantly expressed in E. coli and purified as previously described [47]. EndoS treatment (intravenous, 20 μg) was initiated 1 day before disease induction, before disease onset (day 7), and during the acute phase of the disease (day 14). Control animals received placebo (PBS) injections.

### Analysis of IgG glycan hydrolysis

Mice were bled (tail) on day 1 before immunization, and on days 7 and 14 after immunization. Serum was isolated after coagulation and centrifugation (15 min at 3000 *g*) and kept frozen at −20°C until use. Purification of total IgG in plasma was performed using protein G Sepharose according to the manufacturer’s instructions (GE Healthcare Biosciences). Purified IgG was separated using 10% SDS-PAGE and stained with Coomassie blue (total protein stain), or electroblotted onto polyvinylidene difluoride (PVDF) membranes (immobilon-P; Millipore, Bedford, MA, USA). Equal amounts of material were loaded into each well during SDS-Page analysis. Glycosylated IgG was detected using 1 μg/mL biotinylated *Lens culinaris* agglutinin-A (LCA) lectin and 1 μg/mL of streptavidin-horseradish peroxidase (Vector Laboratories, Burlingame, CA, USA) and SuperSignal West Pico peroxidase substrate (Pierce, Rockford, IL, USA). Membranes were analyzed using a Chemidoc XRS imaging system and Quantity One image analysis software (Bio-Rad, Hercules, CA, USA).

### Serological assay

Serum levels of total IgG were determined by ELISA as described previously [21]. IgG results are expressed in μg/mL by referring to a standard curve obtained using a mouse serum with known IgG concentrations (Miles Laboratories).

### Isolation of CNS-infiltrating mononuclear cells

Mice were anesthetized with sodium thiopental and perfused through the left ventricle with cold PBS until the effluent ran clear. Spinal cords were extruded by flushing the vertebral canal with cold PBS, rinsed in PBS, and minced with a scalpel blade. The spinal cords were forced through 100-mesh stainless steel screens (Falcon, BD Biosciences) to obtain a single-cell suspension in HBSS containing 300 U/mL per cord of type IV clostridial collagenase (Sigma), and were then incubated for 1 h (37°C). The spinal cord homogenate was resuspended in 30% Percoll (Sigma) and underlaid with 70% Percoll. The gradients were centrifuged at 500 *g* at 24°C for 20 min. CNS mononuclear cells were collected from the 30%/70% interface and were washed and resuspended in RPMI 1640 medium (Invitrogen) supplemented with 2 mM L-glutamine, 25 mM HEPES, 100 U/mL penicillin, 100 μg/mL streptomycin, 2-mercaptoethanol (2-ME), and 10% fetal calf serum (FCS) (all supplements from Invitrogen). Typical recovery yielded from 5 × 10^5^ to 1 × 10^6^ cells per spinal cord.

### Isolation of bone marrow cells

Bone marrow cell suspensions were isolated by flushing the femurs and tibias harvested from EndoS- and PBS-treated mice at peak disease. Bone marrow cells were resuspended in complete RPMI 1640 medium (Invitrogen) supplemented with 2 mM L-glutamine, 25 mM HEPES, 100 U/mL penicillin, 100 μg/mL streptomycin, 2-ME, and 10% FCS (all supplements from Invitrogen).

### Spleen cell proliferation assay

Splenocyte cell suspensions were isolated from MOG_35-55_-immunized mice at day 22 by homogenizing spleens through a cell strainer (70 μm Nylon, Falcon®) and removing red blood cells with ACK lysing buffer (BioWhittaker, Walkersville, MD, USA). Splenocytes from six separate mice from the same group were plated in triplicate in 96-well round bottom plates at 2 × 10^5^ cells/well in 200 μL complete RPMI 1640 medium (Invitrogen) supplemented with 2 mM L-glutamine, 25 mM HEPES, 100 U/mL penicillin, 100 μg/mL streptomycin, 2-ME, and 10% FCS (all supplements from Invitrogen) containing either 0 to 50 μg/mL of MOG_35-55_ or CpG (2 μg/mL) and cultured at 37°C, 5% CO_2_ for 72 h. Proliferation was measured by incorporation of ^3^ H-methylthymidine (1 μCi/well) during the last 16 h of culture using a filtermate harvester (Packard Instrument Co.) and a 1450 microbeta liquid scintillation counter (PerkinElmer). Results were determined as mean ± SD from triplicate cultures.

### Immunologic markers and flow cytometry

Cells from spleens, bone marrow, and spinal cords were incubated for 30 min at 4°C with FITC, PE, PerCP-Cy5, or APC fluorochromes conjugated with: CD4, CD8, CD11c, CD11b, CD138, B220, CD69, and CD44. To block non-specific Fc-mediated interactions, cells were incubated in blocking solution (PBS with 1% FCS) for 20 min on ice prior to staining. Samples were run through a FACS Calibur flow cytometer (Becton Dickinson) with standard equipment. Flow cytometry acquisitions were analyzed using FlowJo analysis software (Version 9.3.2).

### Histopathology and immunostaining

Spinal cords at peak of disease (day 22) obtained from transcardially perfused mice (4% paraformaldehyde) were embedded in paraffin (*n* = 4 to 6 animals per experimental group). Paraffin-embedded blocks were cut into 5-μm thick sections and stained with hematoxylin-eosin (HE) to assess inflammation or luxol fast blue/periodic acid Shiff stain (LFB/PAS) to assess the degree of demyelination. Quantification was performed by examining five tranverse sections from the cervical to thoracic spinal cord per mouse, as described previously [48].

For immunohistochemistry, endogenous peroxidases (PBS/3% hydrogen peroxide) were inactivated and unspecific binding was blocked (PBS/10% FCS), followed by incubation with rabbit polyclonal anti-mouse C9 primary antibodies (provided by Dr M Collin, University of Sweden). Bound primary antibodies were visualized using an avidin-biotin technique with 3,3-diaminobenzidine as chromogens (hemalaun counterstaining of nuclei). No significant staining was detected in slides incubated with control rabbit IgG. Numbers of C9-positive deposits within spinal cord lesions/spinal cord white matter (over an area of at least 150 μm × 150 μm per animal) were expressed as C9 density per μm^2^ (density index). Rabbit polyclonal anti-mouse C9 primary antibodies were used to perform the immunofluorescent double staining. The distribution of the primary antibodies was visualized using anti-rabbit IgG secondary antibody conjugated to the rhodamine fluorochrome. DAPI stain was applied to visualize cell nuclei. For negative controls, the primary antibody was replaced with the appropriate non-immune serum. To control for cross-reactivity in double immunofluorescence, sections were incubated with secondary antibody only.

### Western blotting

Spinal cords from EndoS-treated mice and control PBS-treated C57BL6J mice were homogenized using a polytron in lysis buffer (50 mM Tris–HCl, pH 7.5, 250 mM NaCl, 1% Triton X-100, 1 mM EDTA, 1 mM DTT) containing complete protease inhibitors (Roche) and incubated 30 min on ice. Lysates were sonicated and clarified by centrifugation. Protein concentration of the different lysates was determined using Bradford analyses. Aliquots containing equal amounts (20 μg) of total protein or serum were heated to 90°C for 5 min in SDS-sample buffer containing 5% 2-ME (Sigma, Chicago, IL, USA), transferred to a 7.5-12.5% SDS-polyacrylamide gel and blotted on an Immobilon-P PVDF membrane (Millipore, Bedford, MA, USA). After blotting of the proteins, the blocking and antibody incubation steps were performed in BPS containing 5% non-fat milk and 0.1% Tween 20 (Sigma). Complement C1q, C3 (in-house made by Dr S Izui, University of Geneva, Switzerland), and C9 (provided by Dr M Collin, Lund University, Sweden) were detected by incubating PVDF membranes 2 h at room temperature in buffer containing properly diluted goat anti-mouse anti-C1q and anti-C3 antibodies, and rabbit anti-mouse anti-C9 antibody. Binding of the primary antibody was detected using a peroxidase-conjugated secondary antibody to rabbit IgG (Jackson ImmunoResearch labs, West Grove, PA, USA). After washing, positive bands were visualized using chemiluminescence (Supersignal; Pierce, Rockford, IL, USA). Relative protein expression (mean of *n* = 3 animals ± s.e.m.) was quantified using Image J software (NIH).

### Statistical analysis

Data are expressed as mean ± s.e.m. and were analyzed by unpaired two-tailed Student’s *t*-test, using Graphpad Prism 4.01 for Windows (Graphpad Software, San Diego, CA, USA). *P* values ≤0.05 were considered statistically significant.

## Results

### EndoS treatment ameliorates MOG_35-55_-induced EAE in wild-type (not B cell-deficient) mice

**Figure 1:**
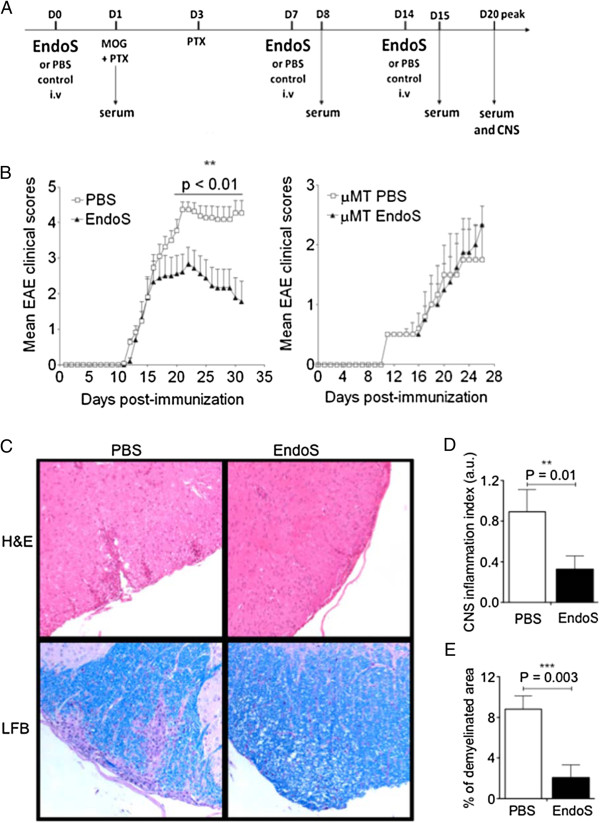
**EndoS treatment ameliorates clinical and histological MOG**_**35-55**_**-induced EAE.** (**A**) A schematic representation of the experimental setup. (**B**) MOG_35-55_-induced EAE is inhibited in WT mice, but not in B cell-deficient (μMT KO) mice, treated with EndoS; EAE scores were determined daily after disease onset in EndoS-treated mice (triangles; *n* = 11) and PBS-treated controls (squares; *n* = 10). Data are presented as mean EAE score ± s.e.m. A significant statistical difference between the two groups of WT mice is observed from peak disease (day 20) to chronic phase (day 30). Data are representative of three individual experiments. (**P < 0.01). (**C**) Representative images of paraffin-embedded spinal cord sections at peak disease (day 22) of indicated groups stained for hematoxylin and eosin (H&E) or Luxol fast blue/Periodic acid Shiff stain (LFB/PAS) (magnification 200x) are shown. Five sections of spinal cord per mouse (*n* = 6 for EndoS-treated mice; *n* = 4 for PBS-treated WT mice) were analyzed, and the number of (**D**) inflammatory lesions and (**E**) extent of demyelination per spinal cord section are presented as histograms. Error bars represent the mean ± s.e.m. **P < 0.01 by Student’s *t*-test.

To evaluate the effect of IgG hydrolysis on MOG_35-55_-induced EAE in C57BL/6 mice, we administered EndoS (20 μg) (*n* = 11) or PBS (*n* = 10) by subcutaneous injection to WT and B cell-deficient mice (μMT KO) 1 day prior to MOG-immunization, before disease onset (day 7), and during the acute disease phase (day 14) (Figure 1A). PBS-treated WT mice developed severe, chronic, progressive EAE, while disease severity was significantly diminished in WT mice treated with EndoS (Figure 1B, left panel). In WT mice, EndoS treatment alleviated the severity of EAE during the acute phase (day 15 post-immunization) and impeded the full development of EAE during the chronic phase (from day 22 to 32 post-immunization) even though treatment was discontinued (Figure 1B). In contrast to WT mice, the positive treatment effect of EndoS in EAE was abrogated in μMT KO mice, confirming that the beneficial effect of EndoS in MOG_35-55_-induced EAE requires B cells or B cell products (Figure 1B, right panel).

### EndoS suppresses pathological signs of EAE

Consistent with the clinical finding, histological examination of CNS tissues revealed a significant pathological difference between EndoS- and PBS-treated WT mice at the disease peak (Figure 1C). Figure 1D and 1E depict inflammation and demyelination scores from WT mice that received PBS or EndoS and were sacrificed on day 22 post-immunization. In PBS-treated WT mice, multiple inflammatory foci were observed in the white matter of the spinal cord, together with a high degree of demyelination. By contrast, significantly fewer inflammatory lesions and little demyelination were detected in EndoS-treated WT mice.

### Endoglycosidase treatment efficiently hydrolyzes IgG glycans *in vivo* in EAE mice

**Figure 2:**
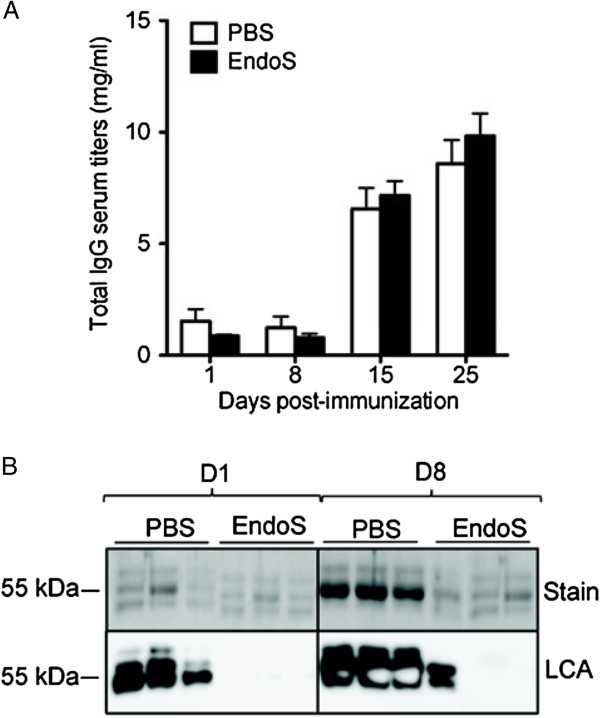
**Endoglycosidase treatment has a profound effect on IgG glycan hydrolysis*in vivo*.** (**A**) Total serum IgG levels isolated from EndoS-treated WT mice (*n* = 6) and PBS-treated treated WT mice (*n* = 4) at the indicated time after immunization were determined by ELISA. (**B**) SDS-PAGE Coomassie Blue staining (stain) and lectin blot analysis (LCA) of purified IgG from serum samples drawn from three representative PBS- or EndoS-treated WT mice at indicated times after intravenous injection of 20 μg EndoS or PBS (1 and 8 days).

To investigate the IgG glycan-hydrolyzing activity of EndoS in the circulation of live animals, C57BL6/J WT mice were intravenously injected three times (on days 0, 7, and 14) with PBS or 20 μg of EndoS, and serum IgG samples (drawn on days 1, 8, 15, and 25) were analyzed for glycosylation status using sodium dodecyl sulfate polyacrylamide gel electrophoresis (SDS-PAGE) and lectin blot analyses using *Lens culinaris* agglutinin-A (LCA blots). We first demonstrated that systemic EndoS treatment did not affect IgG antibody titers during the acute and chronic phases of EAE (Figure 2A). As previously reported [21], we observed that IgG levels tightly coincided with increased disease severity, reaching 6.56 ± 0.5 mg/mL (mean ± s.e.m.) at day 15 and 8.59 ± 0.53 mg/mL at day 25 post-immunization, compared with 1.516 ± 0.28 mg/mL before disease onset in PBS-treated mice (*P* < 0.0001, Student’s *t*-test) (Figure 2A). One day after EndoS injection, the apparent molecular mass of the serum IgG heavy chain from EndoS-treated mice revealed a complete shift toward an approximate 3-kDa smaller protein band, compared with fully glycosylated intact mouse IgG from PBS-treated mice (Figure 2B, stain blot). The apparent lack of correlation between serum IgG levels (Figure 2A) and the heavy chain signal (Figure 2B, stain blot) is likely to reflect the different sensitivities of the two assays. Lectin blot analysis revealed that the serum IgG heavy chain carbohydrate signal was abolished one day after EndoS injection, further confirming complete IgG glycan hydrolysis (Figure 2B, LCA blot). The IgG glycan hydrolysis patterns were nearly identical after injections 1 and 2, compared with mice that had not previously been exposed to EndoS (Figure 2B), with the exception of one animal that exhibited partial hydrolysis of IgG by EndoS on day 8. 

### EndoS treatment does not influence peripheral or central immune cell function during EAE

**Figure 3:**
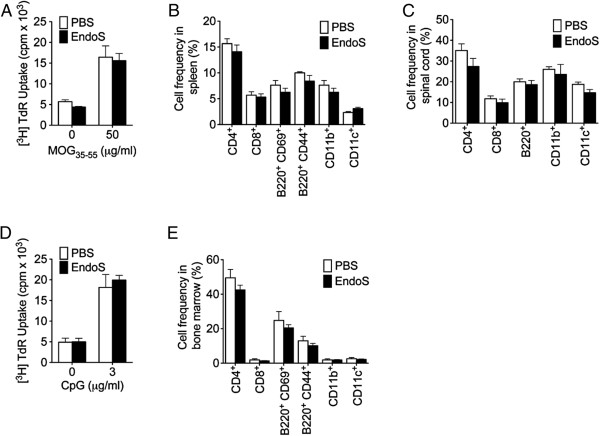
**EndoS treatment does not limit T cell activation, B cell activation, or CNS recruitment of lymphoid or myeloid cells.** (**A**) EndoS has no impact on peripheral T cell priming or recall responses of spleen cells in response to MOG_35-55_ peptide, as measured using a ^3^ H-thymidine incorporation assay. EndoS treatment does not influence the (**B**) peripheral frequency or activation or (**C**) CNS (spinal cord) infiltration of important lymphoid and myeloid immune cell populations, as measured (mean ± s.e.m.) by flow cytometry analysis of cells isolated from EndoS-treated mice (*n* = 6) and PBS-treated mice (*n* = 4) at day 25 post-immunization. (**D**) CpG is equally effective in stimulating the proliferation of splenic B cells from PBS- or EndoS-treated mice, as determined by ^3^ H-thymidine incorporation after 3 days of culture. (**E**) EndoS treatment does not affect the frequency or activation status of immune cells in the bone marrow.

Considering the fact that CD4^+^ T cells are initiators of EAE and drivers of neuro-immune degeneration in the CNS, we investigated whether EndoS treatment would interfere with their cellular functions. We observed that EndoS treatment did not block the T-cell recall response to the encephalitogenic peptide MOG_35-55_, suggesting that EndoS had no impact on peripheral priming of MOG-specific autoreactive T cells (Figure 3A). In support of this assumption, we observed no difference in T cell (CD4^+^ and CD8^+^) peripheral frequency (Figure 3B) or recruitment to the CNS between PBS- and EndoS-treated EAE mice (Figure 3C). Likewise, CpG-dependent B cell proliferation (Figure 3D), peripheral frequency of CD44^+^ or CD69^+^ activated B cells (Figure 3B), and B cell recruitment into the CNS (Figure 3C) were not affected by EndoS treatment. In addition, we did not observe any difference in the peripheral or CNS frequency of myeloid cells, such as macrophages (CD11b^+^) and dendritic cells (CD11c^+^), prominent constituents of neuroinflammatory infiltrates in the CNS during EAE that have the capacity to activate T cells (Figure 3B,C). Finally, EndoS treatment did not affect the frequency or activation status of immune cells in the bone marrow (Figure 3E).

### EndoS treatment impairs IgG-dependent complement activation and consumption during MOG_35-55_-induced EAE

**Figure 4:**
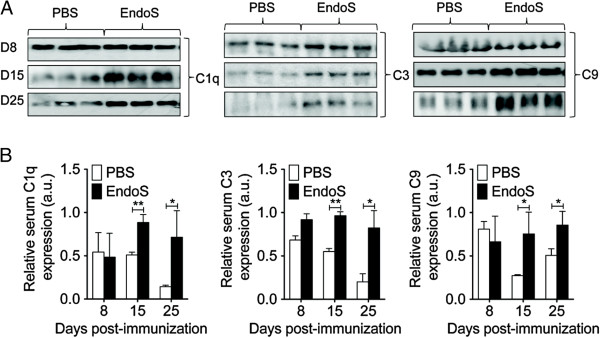
**EndoS inhibits peripheral complement activation in EAE mice.** (**A**) Serum complement activation or consumption of complement components C1q, C3, and C9 was evaluated by SDS-PAGE analysis of sera from EndoS- and PBS- treated WT mice (*n* = 3 per group) at the indicated time after immunization. (**B**) Relative serum expression of complement components C1q, C3, and C9 (*n* = 3 independent mice per group) was expressed as arbitrary units of density of bands (intensity/mm^2^).

To determine whether the mechanism by which EndoS protection involves the modulation of complement activation, we studied changes in complement component (C1q, C3, and C9) consumption in EndoS-treated and untreated WT mice by SDS-PAGE analysis at different stages of the disease progression. As previously reported, EndoS hydrolysis of IgG glycan decreased serum complement activation as early as day 15 post-immunization in MOG_35-55_-induced EAE mice (Figure 4A,B). EndoS treatment had no effect on serum complement consumption at day 8 post-immunization, further confirming the absence of complement-activating IgG antibodies (Figure 2A) at the onset of EAE.

### Complement component deposition is reduced in the CNS by EndoS in EAE mice

**Figure 5:**
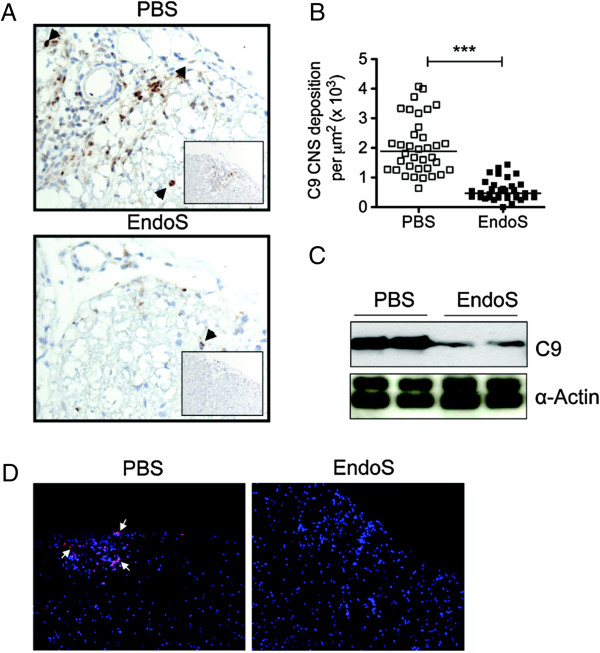
**High levels of C9 are detected in the spinal cord of PBS-treated EAE mouse models, but absent from EAE mice treated with EndoS.** (**A**) Representative C9 immunostaining (brown) in the spinal cord of WT MOG_35-55_ peptide-induced EAE mice at day 22 post-immunization (disease peak) (magnification 200x). (**B**) EndoS treatment significantly lowered the density of C9 deposits in mouse spinal cord (*n* = 4). ***, *P* ≤0.001 by Student’s *t*-test. (**C**) A representative western blot of spinal cord extracts from EndoS- and PBS-treated mice (*n* = 3 per group) probed with the anti-C9 antibody. Anti-α-Actin served as the loading control. (**D**) Complement C9 deposition in the CNS is strongly reduced by EndoS treatment in EAE mice. Double-label immunofluorescence microscopy demonstrated that complement component C9 (pink-purple) is deposited in the CNS of PBS-treated (but not EndoS-treated) mice. Cell nuclei were counterstained with DAPI (blue).

To investigate whether EndoS treatment influences CNS complement deposition during MOG_35-55_-induced EAE, spinal cords from EndoS- and PBS-treated mice were examined at day 22 post-immunization (peak disease) for immunohistochemical detection of complement component C9. We observed a significant reduction of C9 deposit density in EndoS-treated mice (0.58 ± 0.06 × 10^3^/μm^2^, *n* = 35 lesions) compared with PBS-treated control mice (2.05 ± 0.16 × 10^3^/μm^2^, *n* = 36 lesions) (Figure 5A,B). Consistent with this observation and peripheral C9 complement activation, C9 was detected by western blotting and immunofluorescence microscopy in spinal cord from PBS-treated, but not EndoS-treated, WT mice (Figure 5C,D). These data suggest both a role for C9 in brain inflammation during MOG_35-55_-induced EAE, and a possible inhibitory mechanism of action of EndoS.

## Discussion

EAE shares clinical and histopathological similarities with MS and serves as the most widely used animal model for the disease. Data from MS patients and EAE studies support a role for B cells/antibodies in the disease process. The presence of oligoclonal bands in the cerebrospinal fluid (CSF) of MS patients, and their association with a worse prognosis, supports a pathogenic role for antibodies in CNS autoimmunity [49,50]. Here, we established that enzymatic IgG glycan hydrolysis by EndoS attenuates MOG_35-55_-induced EAE. We observed no differences in CNS-infiltrating CD4^+^ T cell frequencies or activation during EAE, confirming that EAE protection occurs independently of T cell immunity. Clinical benefit of EndoS treatment in mice was associated with reduced complement system activation in the presence of comparable IgG levels between treated and untreated groups, supporting a profound effect of IgG glycan on IgG effector functions. Overall, our data strengthen the importance of antibodies in MOG_35-55_-induced EAE, a model of CNS inflammation that is considered less dependent upon B cells and humoral immune responses, and also suggest that the EndoS enzyme could be used as an immunomodulatory therapeutic agent against IgG-mediated CNS autoimmune diseases.

Although the role of CD4^+^ T cells in EAE/MS initiation is widely accepted, the role of B cells and antibodies in disease pathogenesis remains unclear. Several previous studies have however provided cumulative evidence for an active role of anti-myelin antibodies in inducing demyelination [7,8,11]. In EAE, although anti-MOG antibodies cannot trigger CNS inflammation on their own, they strongly augment T cell-initiated and macrophage-enhanced demyelination, and may augment disease severity [51,52]. Several studies indicate that the demyelinating activity of myelin-specific antibodies is related to their ability to fix complement [53]. Other roles for B cells in MS/EAE pathogenesis include directing the polarization, activation, or specificity of disease-relevant T cells [13,54], production of pro- and anti-inflammatory cytokines, and potentiation of remyelination [55].

Early studies in the C57BL/6 mouse EAE model have indicated that the induction of disease may be B cell-dependent, depending on the source and nature of the MOG immunogen. Induction of EAE after immunization with MOG_35-55_[17,25] or recombinant MOG (rMOG) protein from mouse or rat [17,56] but not human [25,56] was shown to be B cell-independent. However, recent B cell depletion studies and other experimental paradigms have demonstrated both protective and pathogenic roles for B cells in EAE models in which they were previously considered to contribute only marginally to disease pathogenesis [13,14,17]. Previous data from the EAE models indicated that the role of antibodies in the disease process might differ based on the model employed [25,56]. While immunization with rodent or human rMOG [56] or MOG_35-55_[57] leads to the development of antibodies that bind mouse MOG, reports indicate that only MOG-specific antibodies generated in response to human rMOG are pathogenic [25]. However, recent cumulative data support a pathogenic role for antibodies in MOG_35-55_-induced EAE in C57BL/6 mice [18-21].

In this study, we report that specific IgG glycan hydrolysis by EndoS attenuates MOG_35-55_-induced EAE independent of T cell immunity. Importantly, we observed a clear association between the appearance of serum antibodies and the occurrence of clinical EAE signs. In this context, we found that the beneficial effect of EndoS in neutralizing the effector functions of antibodies was only evident shortly after disease onset, when myelin-specific antibodies are thought to contribute to disease progression. Additionally, our findings demonstrating a reduction of complement activation, and in particular C9, after EndoS administration are strongly suggestive of a role for this system in the development and clinical expression of MOG_35-55_-induced EAE. In addition to the complement system, it is also conceivable that EndoS might interfere with a function of FcR in antibody-mediated demyelination [58,59], such as inefficient ADCC [60]. However, this concept is strongly challenged by EAE studies indicating that autoantibody-directed demyelination depends on complement activation but not activatory FcγR [33,59] and that the antibody-binding FcαR units are not required for disease development [61].

Several reports indicate that complement activation may play an essential role in the mediation and maintenance of inflammatory reactions and the process of demyelination in the CNS [62], particularly in diseases such as MS and neuromyelitis optica (NMO). Terminal complement component has been shown in actively demyelinating areas of the CNS in MS [5,63], and evidence exists that complement (specifically, the assembly of the C5b-9 complex) promotes myelin damage and demyelination in well myelinated mouse cerebellum cultures [64]. Increased levels of the C5b-9 complex have been detected in the spinal fluid of MS patients during relapses [65,66], and these levels have been correlated with neurological disability. In EAE, several studies have used experimental manipulations that reduce the activation of the complement to evaluate its role in disease pathogenesis. Among others, serum complement depletion with cobra venom factor (CVF) reduced actively induced acute EAE in rats [62]. Similar to our findings, CVF and its effect on the complement cascade have been shown to reduce the severity of EAE-related inflammation and demyelination without significantly influencing certain histopathological concomitants [23,62]. In support of our findings, activation of C9 (the major component of the cytolytic membrane attack complex) in EAE is related to myelin injury rather than CNS inflammation [67].

While our data indicate that reduced complement activation by EndoS hydrolysis of IgG may account for its clinical benefit in EAE, recent findings suggest that EndoS-hydrolyzed human IgG does bind better to FcγRIIb than non-hydrolyzed IgG under certain circumstances [68]. In the context of MOG_35-55_-induced EAE pathogenesis, this finding might be relevant, as recent evidence suggest that autoimmune-prone FcγRIIb-deficient mice exhibit more pronounced MOG_35-55_-induced EAE disease [60]. Importantly, intravenous Ig (IVIG) injection, which is reportedly therapeutic in MOG_35-55_-induced EAE [69] and has exhibited some evidence of efficacy in MS [70], may act by targeting the low-affinity IgG inhibitory receptor FcγRIIb [71]. Therefore, one could speculate that EndoS treatment in MOG_35-55_-EAE may have a dual anti-inflammatory activity, both inhibiting the activation of the complement and shifting the binding of pathogenic IgG toward the inhibitory action mediated through FcγRIIb. Although additional studies are needed to test this hypothesis, it represents a potential additional mechanism of action of EndoS that could account for its protective effects in EAE. Moreover, while data indicate that EndoS specifically hydrolyzes native IgG (no other substrate has been identified to date) [47], we could not exclude the possibility that EndoS could cleave glycans other than those on IgG. Whether IgG deglycosylation plays a more significant role in a B cell-dependent EAE model using recombinant MOG protein would therefore be of specific interest.

The presence of Ig’s and complement activation products in active MS lesions and the efficacy of therapeutic plasma exchange in some patients provide circumstantial evidence for the involvement of antibodies in MS [72,73]. Here, we establish that administration of EndoS, an enzyme from *Streptococcus pyogenes* that efficiently hydrolyzes the functionally important and conserved N-linked glycan of IgG *in vivo*, significantly attenuates disease progression in a model of CNS inflammation and demyelination. Together with treatment with B cell-depleting antibodies that target cellular function of B cells [74], we propose that EndoS-mediated IgG hydrolysis may constitute a potential novel therapeutic principle for the treatment of IgG-driven human CNS autoimmune diseases such as MS. Reasonable pharmacokinetics of this enzyme is further ensured by its ability to avoid antibody-mediated elimination [42] via removal of glycans necessary for the binding of neutralizing antibodies.

## Competing interests

Patents for the *in vitro* and *in vivo* use of EndoS have been applied for by Genovis AB and Hansa Medical AB, respectively. MC is listed as an inventor on these applications that are pending. MC is a part time scientific consultant for Hansa Medical AB.

## Authors’ contributions

MB, NM, M-LS-R, and PHL designed research; MB, NM, MC, and DM performed research; MC, M-LS-R, and MSW contributed new reagents or analytic tools; MB, NM, M-LS-R, and PHL analyzed data; MB, NM, and PHL wrote the paper. All authors read and approved the final manuscript.
